# Clinical effect of dexmedetomidine combined with sufentanil on postoperative analgesia for transthoracic device closure of ventricular septal defects in children with ultrafast track anesthesia

**DOI:** 10.1186/s13019-021-01592-x

**Published:** 2021-07-28

**Authors:** Jing Wang, Wen-Peng Xie, Yu-Qing Lei, Zeng-Chun Wang, Hua Cao, Qiang Chen

**Affiliations:** 1 Department of Cardiac Surgery, Fujian Maternity and Child Health Hospital, Affiliated Hospital of Fujian Medical University, Fuzhou, China; 2 Fujian Branch of Shanghai Children’s Medical Center, Fuzhou, China; 3 Fujian Children’s Hospital, Fuzhou, China; 4 Fujian Key Laboratory of Women and Children’s Critical Diseases Research, Fujian Maternity and Child Health Hospital, Fuzhou, China

**Keywords:** Dexmedetomidine, Sufentanil, Fast-track anesthesia, VSD

## Abstract

**Background:**

To observe the effect of combining dexmedetomidine with sufentanil on postoperative analgesia in children who underwent transthoracic device closure of ventricular septal defects (VSDs) with ultrafast track anesthesia.

**Methods:**

This was a retrospective study. Eighty-seven children who underwent transthoracic device closure of VSDs were retrospectively analyzed. Patients were divided into three groups based on the different drugs used for postoperative patient-controlled analgesia.

**Results:**

No statistically significant differences in hemodynamic parameters were noted among the three groups after surgery (*p* > 0.05). The FLACC score in the SD2 group was significantly greater than those in the S groups and SD1 groups after surgery (*p* < 0.001). The Ramsay score in the S group was significantly lower than that of the SD1 and SD2 groups at 6 h (*p* < 0.001 and *p* = 0.003), 12 h (*p* = 0.002 and *p* = 0.012), and 24 h (*p* < 0.001 and *p* < 0.001) after surgery. The pressing frequency of the analgesic pump 48 h after the operation in the SD2 group was significantly greater than that in the other two groups (*p* < 0.05). The incidences of respiratory depression, nausea, and vomiting in the S group were significantly greater than those in the SD1 and SD2 groups (*p* < 0.05).

**Conclusion:**

The combination of 0.04 μg/kg/h dexmedetomidine and 0.04 μg/kg/h sufentanil intravenous analgesia was more effective than the other two analgesic strategies in children who underwent transthoracic device closure of ventricular septal defects (VSDs) with ultrafast track anesthesia.

## Background

With the rapid development of minimally invasive cardiac surgery techniques, echocardiography-guided transthoracic device closure has been widely used in the treatment of VSD. This technique has the merits of a small incision, short operative time, quick postoperative recovery, and fewer complications [1, 2]. Ultrafast track anesthesia refers to selecting lower opioid dosing or short-acting opioids to achieve the aim of removing tracheal intubation immediately or within 1 h after cardiac surgery [3], which reduces complications, shortens the length of intensive care unit (ICU) and hospital stay, and optimizes medical resources [4–6]. Ultrafast track anesthesia technology is more conducive to promoting postoperative recovery. However, it also highlights the problem of postoperative pain. Behavioral research has shown that poor pain control has adverse effects on children [7–9].

Intravenous analgesia is generally used in postoperative analgesia, and opioids, such as sufentanil, are used as the primary drug. However, opioids are associated with an increased possibility of nausea, vomiting, and even respiratory depression. Dexmedetomidine has analgesic, sedative, anti-anxiety, and sympathetic nervous system effects. Our systematic review and meta-analysis found that dexmedetomidine combined with sufentanil used in patient-controlled intravenous analgesia (PCIA) improves patient satisfaction and did not increase the incidence of adverse reactions [10]. However, there have been no studies on dexmedetomidine application combined with sufentanil for intravenous analgesia in children who underwent transthoracic device closure of VSDs with ultrafast track anesthesia to date. We hypothesized that dexmedetomidine combined with sufentanil reduces postoperative pain in those children. This study aimed to investigate the postoperative analgesic effect and the side effects of sufentanil of these two drugs in children who underwent transthoracic device closure of VSDs with ultrafast track anesthesia.

## Methods

A retrospective, observational study design was used in this study. We analyzed the clinical data of 87 children who experienced transthoracic device closure of VSDs with ultrafast track anesthesia between June 2019 and June 2020. The inclusion criteria were as follows: (1) children who completed transthoracic device closure of VSDs; and (2) the ultrafast track anesthesia plan was used. Cardiac function and hemodynamics were stable after the operation. Children were administered intravenous analgesia after the operation using sufentanil and/or dexmedetomidine. The exclusion criteria were as follows: (1) failure to complete transthoracic device closure of VSDs; and (2) liver and kidney dysfunction or other vital organ dysfunction.

All patients were informed of the postoperative analgesia strategy, and different analgesia strategies were provided to patients according to different doctors’ medication preferences. We divided the patients into three groups based on the drugs used for postoperative patient-controlled intravenous analgesia: the S group (sufentanil 0.05 μg/kg/h, n = 28), SD1 group (dexmedetomidine 0.04 μg/kg/h and sufentanil 0.04 μg/kg/h, n = 30) and SD2 group (dexmedetomidine 0.04 μg/kg/h and sufentanil 0.03 μg/kg/h, n = 29). Although the groups were not double-blinded, subsequent ICU doctors and staff treated them according to the same criteria.

After routine preoperative fasting for 4–6 h and fluid deprivation for 2–4 h, the patients received basal anesthesia with 0.5 mg/kg oral midazolam. Anesthesia induction was conducted by intravenous injection of 0.3 mg/kg etomidate, 1.5 μg/kg remifentanil, and 0.9 mg/kg rocuronium. Mechanical ventilation was performed using the pressure control mode following tracheal intubation. Anesthesia maintenance was performed by injection of 0.5–1 μg/kg/min remifentanil and inhalation of 2–3% sevoflurane. The depth of anesthesia was maintained by regulating the dose of the drug during the operation. Blood pressure, oxygen saturation, central venous pressure, electrocardiogram, body temperature, blood gas, and electrolytes were monitored continuously to recognize and solve problems promptly. At the end of the operation, the children recovered spontaneous breathing with body movements, head up, eyes open, and normal coughing reflex. Then, the tracheal tube was removed. The patients were transferred to the cardiac ICU.

An Apon intravenous electronic analgesia pump (Jiangsu Apon Medical Technology Co., Ltd.) was used for intravenous analgesia administration under the patient-controlled analgesia (PCA) model. The following configurations of the analgesic pump were used: 0.05 μg/kg/h sufentanil in group S, 0.04 μg/kg/h dexmedetomidine and 0.04 μg/kg/h sufentanil in groups SD1, and 0.04 μg/kg/h dexmedetomidine and 0.03 μg/kg/h sufentanil in group SD2. All three groups of drugs were added to physiological saline at a total volume of 100 ml, and an analgesic pump was used for 48 h after surgery. The analgesic pump was administered with the routine infusion rate (2 ml/h). When the patient experienced severe pain with a FLACC (Face, Legs, Activity, Cry, Consolability) score ≥ 4, the ICU physicians and nurses increased the dosage or gave an additional PCA press (the pressing dose was 1 ml).

The heart rate (HR), mean arterial pressure (MAP), FLACC score, and Ramsay score of the patients at 2 h (T1), 6 h (T2), 12 h (T3), 24 h (T4), and 48 h (T5) after the operation were recorded. The FLACC pain scale was used to evaluate the analgesic effect [11]. We measured each behavior on a 0–2 scale. Here, 0 points indicates relaxation and comfort, 1–3 points indicates slight discomfort, 4–6 points indicates moderate pain, and 7–10 points indicates severe pain and discomfort. The Ramsay score was used to evaluate the sedative effect [12] based on the following scoring system: (1) patient anxious, agitated or restless or both; (2) patient cooperative, oriented, tranquil, and alert; (3) patient responds to commands; (4) asleep, but with a speedy response to a light glabellar tap or loud auditory stimulus; (5) asleep, sluggish response to a light glabellar tap or loud auditory stimulus; and (6) asleep, shows no response to a light glabellar tap or loud auditory stimulus. A score of 1 point indicated a lack of sedation, 2–4 points indicated appropriate sedation, and 5–6 points indicated excessive sedation.

### Statistical analysis

We used SPSS 25 software to perform statistical analysis. Continuous data are presented as the mean ± standard deviation and range. ANOVA was used to compare the mean of the three groups, and the S–N–K(S) method was used to compare each pair. The χ^2^ or Fisher’s test was employed to classify variables. Non-parametric data are presented as medians, and differences were evaluated using the Kruskal–Wallis H test. A *p* value of < 0.05 was regarded as statistically significant.

## Results

No significant differences in general preoperative information or operation time were noted among the three groups, indicating that the three groups were homogeneous and comparable (*p* > 0.05). (Table 1) The hemodynamic parameters among the three groups at 2 h, 6 h, 12 h, 24 h, and 48 h after surgery were not significantly different (*p* > 0.05) (Table 2).

**Table 1:** Demographic and clinical characteristics of the three groups (x ± s)

	S group	SD1 group	SD2 group	*p* value
Number	28	30	29	
Male/female	15/14	14/15	14/15	0.861
Age (year)	3.3 ± 1.3	3.2 ± 1.1	3.4 ± 1.0	0.862
BMI	18.9 ± 1.5	18.7 ± 2.1	19.2 ± 2.3	0.613
Operation time (min)	43.5 ± 10.2	46.3 ± 8.8	44.5 ± 11.1	0.561
Ventricular septal defect size (mm)	4.3 ± 1.2	4.3 ± 1.3	4.2 ± 1.1	0.672
Pulmonary arterial pressure (mmHg)	25.2 ± 5.1	22.2 ± 6.9	23.2 ± 7.3	0.524
Occluder size (mm)	5.3 ± 1.1	5.2 ± 1.2	5.5 ± 1.0	0.823

**Table 2:** The comparison of perioperative hemodynamics, FLACC score and Ramsay score of the three groups (x ± s)

	S group	SD1 group	SD2 group	F value	*p* value
T1					
MAP (mmHg)	55.32 ± 5.26	55.13 ± 4.09	56.72 ± 4.11	1.083	0.343
HR (bpm)	110.18 ± 8.06	113.17 ± 8.71	112.07 ± 10.05	0.815	0.446
T2					
MAP (mmHg)	52.62 ± 3.75	54.47 ± 3.58	52.55 ± 4.36	2.254	0.111
HR (bpm)	115.64 ± 9.75	115.90 ± 6.83	116.45 ± 10.84	0.057	0.945
T3					
MAP (mmHg)	56.64 ± 4.89	55.87 ± 4.69	56.03 ± 4.90	0.205	0.815
HR (bpm)	110.46 ± 6.87	111.57 ± 7.53	109.00 ± 8.34	0.843	0.434
T4					
MAP (mmHg)	51.82 ± 4.71	53.3 ± 3.29	51.55 ± 3.47	1.760	0.178
HR (bpm)	105.39 ± 8.43	105.60 ± 7.63	107.55 ± 6.74	0.706	0.497
T5					
MAP (mmHg)	50.46 ± 5.22	50.67 ± 4.80	52.10 ± 3.20	1.147	0.332
HR (bpm)	107.57 ± 8.47	104.93 ± 9.14	105.76 ± 7.11	0.761	0.470

MAP, the mean arterial pressure; HR, the heart rate

**Fig. 1:**
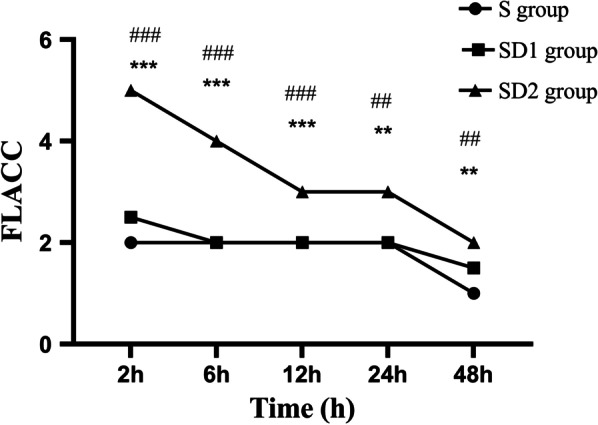
FLACC score observed at each time point for all patients (***indicates that compared with the SD1 group, *p* < 0.001; ^###^indicates that compared with the S group, *p* < 0.001; **indicates that compared with the SD1 group, *p* < 0.05; ^##^indicates that compared with the S group, *p* < 0.05)

The FLACC score in the SD2 group was significantly greater than that in the S group and SD1 group at 2 h (*p* < 0.001 and *p* < 0.001), 6 h (*p* < 0.001 and *p* < 0.001), 12 h (*p* < 0.001 and *p* < 0.001), 24 h (*p* = 0.002 and *p* = 0.009), and 48 h (*p* = 0.005 and *p* = 0.008) after surgery. However, no statistically significant differences in the FLACC score were noted between the S and SD1 groups at each time point (*p* > 0.05). (Fig. 1).

**Fig. 2:**
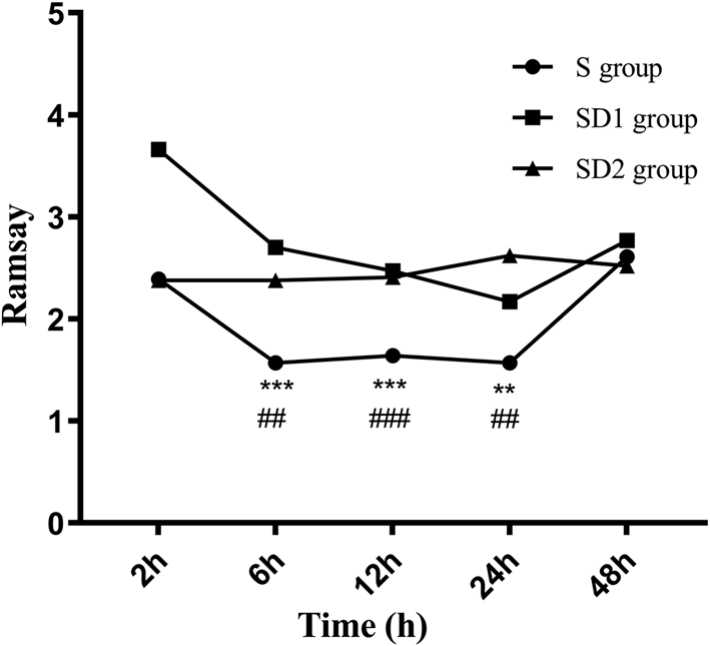
Ramsay score observed at each time point for all patients (***indicates that compared with the SD1 group, *p* < 0.001; ^###^ indicates that compared with the S group, *p* < 0.001; **indicates that compared with the SD1 group, *p* < 0.05; ^##^indicates that compared with the S group, *p* < 0.05)

The Ramsay score in the S group was significantly lower than those in the SD1 group and the SD2 group at 6 h (*p* < 0.001 and *p* = 0.003), 12 h (*p* = 0.002 and *p* = 0.012), and 24 h (*p* < 0.001 and *p* < 0.001) after surgery, but no significant difference was noted between the SD1 group and the SD2 group (*p* > 0.05). No statistically significant differences in the Ramsay score at 2 h (*p* = 0.609) and 48 h (*p* = 0.615) after surgery (*p* > 0.05) were noted among the three groups. (Fig. 2).

The pressing frequency of the analgesic pump 48 h after surgery in the SD2 group was significantly greater than that in the S group and the SD1 group (*p* < 0.05). The incidences of respiratory depression, nausea, and vomiting in the S group were significantly greater than those in the SD1 and SD2 groups (*p* < 0.05) (Table 3).

**Table 3:** Comparison of postoperative complications of the three groups

Parameter	S group	SD1 group	SD2 group	*p* value
Pressing frequency	5.1 ± 1.2	4.6 ± 1.0	10.5 ± 3.1*^#^	0.011
Nausea and vomiting: case (%)	6(21.43)	1(3.33)*	1(3.44)*	0.025
Bradycardia: case (%)	0(0)	0(0)	0(0)	–
Respiratory depression: case (%)	3(10.71)	0(0)*	0(0)*	0.038
Excessive sedation: case (%)	0(0)	0(0)	0(0)	–

*Indicated that compared with group S, *p* < 0.05; ^#^indicated that compared with group SD1, *p* < 0.05

## Discussion

Transthoracic device closure is a commonly used and effective minimally invasive method to treat patients with restrictive VSD in China and has the merits of not requiring cardiopulmonary bypass, the lack of X-ray exposure, a small surgical incision, and a short operation time. However, a small incision is made in the chest. Although ultrafast track anesthesia could decrease the doses of long-lasting opioids during the operation, the patient’s postoperative pain did not seem to abate. Studies have found that pain at an early age affects children’s brain development and changes cognition and immune function [13]. Through a questionnaire survey, Lauridsen et al. found that the prevalence of chronic pain in children after sternotomy was very low, but it should not be ignored [14]. Analgesia treatment after cardiac surgery needs to be given sufficient attention and treated actively. In a patient undergoing ultrafast track anesthesia, the tracheal tube will be removed early, which requires professional sedation and pain control management by the anesthesiologist to ensure normal spontaneous breathing and the patient’s comfort and to promote fast recovery [15].

Patient-controlled analgesia produces a stable blood concentration, which is often used for postoperative analgesia in children, and its effectiveness has been well documented [16, 17]. Sufentanil is the opioid with the most potent analgesic effect, and its analgesic strength is 1000 times that of morphine and 7–10 times that of fentanyl. Sufentanil has been widely used in pediatric anesthesia and postoperative analgesia. However, to achieve the best analgesic effect, the use of sufentanil alone requires a large dose, quickly causing adverse reactions to opioid drugs, such as nausea, vomiting, and even respiratory depression. As a highly selective α-2 adrenergic agonist, dexmedetomidine exhibits hypnotic, sedative, analgesic, and anxiolytic properties. More importantly, dexmedetomidine is not associated with respiratory depression. Therefore, this study retrospectively investigated the analgesic effect and the side effects of sufentanil combined with dexmedetomidine as a postoperative analgesia strategy for children who undergo transthoracic device closure VSD with ultrafast track anesthesia.

In this study, the FLACC scores in the S group and the SD1 group at each time point after surgery were all less than 4, suggesting that these two treatment options could provide adequate analgesia. The FLACC score in the SD2 group was significantly greater than those in the S group and the SD1 group at each time point after surgery. Patients in the SD2 group had a significantly more pressing analgesic pump frequency 48 h after the surgery. These results indicated that the analgesic effect in the SD2 group was not as satisfactory as that in the S group and SD1 group, so the pressing frequency of PCA increased to meet adequate analgesia in the SD2 group. The S group had a higher incidence of respiratory depression, nausea, and vomiting. We considered that the pharmacokinetics of sufentanil caused such results. The greater the sufentanil dose, the greater the effect on respiratory depression. A large dose of sufentanil was administered in the S group, so it had a good analgesic effect. Simultaneously, it caused side effects in children, such as respiratory depression, nausea, and vomiting. The total amount of sufentanil was reduced in the SD1 and SD2 groups based on the addition of dexmedetomidine. Other studies have also confirmed that dexmedetomidine combined with opioids in PCA could reduce postoperative opioid demand and opioid-related adverse events [18, 19]. Song and his team found that the use of fentanyl combined with dexmedetomidine for PCA reduces postoperative nausea frequency and severity, which is consistent with our results [20].

The Ramsay score of the S group 6 h, 12 h, and 24 h after surgery was significantly lower than those of the SD1 group and the SD2 group at. This finding could be explained by the fact that the S group was not compounded with dexmedetomidine, whereas 0.04 μg/kg/h dexmedetomidine in the SD1 and SD2 groups exerted a sedative effect. Dexmedetomidine converges on an endogenous nonrapid eye movement sleep-promoting pathway to exert its sedative effects [21]. In this study, the three groups of children’s hemodynamic indexes were stable, indicating that all three regimens were well tolerated in all patients. The most common side effects of dexmedetomidine were hypotension and bradycardia [22], which did not occur in any group in our study. This finding is potentially explained by the limited number of patients included in this study and that the pooled prevalence incidence of bradycardia was 2.6% [23]. Thus, so no adverse reactions of bradycardia were found. Studies have also suggested that using a lower dose could avoid dexmedetomidine’s hemodynamic effects, and continuous infusion of dexmedetomidine would reduce the occurrence of hypotension [24–26].

## Limitation

This research had some limitations. No unified strategy or literature is available to provide guidance on the compatibility ratio of the two drugs. We adopted the strategy often used in our center, which might affect the accuracy of the results, and such results need to be further assessed. The sample size included in this study was relatively small and was limited to children who underwent transthoracic device closure of VSDs, so it might be unreasonable for our results to be applied to other postoperative patient groups. Our data collection might be biased. Only a few indicators were adopted in the study, and the data of this study might be biased based on the recording process, which might influence the precision of the results. In addition, this was a retrospective study rather than a prospective randomized controlled study, limiting the statistical potency to some extent. Future research needs to take into account the above various factors.

## Conclusion

The combination of 0.04 μg/kg/h dexmedetomidine and 0.04 μg/kg/h sufentanil intravenous analgesia was more effective and resulted in fewer adverse reactions than the other two analgesic strategies in children who underwent transthoracic device closure of ventricular septal defects (VSDs) with ultrafast track anesthesia.

## Data Availability

The data sets used and/or analyzed during the current study are available from the first author or the corresponding author on reasonable request.

## Abbreviations

VSD: Ventricular septal defect
ICU: Intensive care unit
PCIA: Patient-controlled intravenous analgesia
